# A meta-analysis of MSI frequency and race in colorectal cancer

**DOI:** 10.18632/oncotarget.8945

**Published:** 2016-04-23

**Authors:** Hassan Ashktorab, Sadhna Ahuja, Lakshmi Kannan, Xavier Llor, Nathan A. Ellis, Rosa M. Xicola, Adeyinka O. Laiyemo, John M. Carethers, Hassan Brim, Mehdi Nouraie

**Affiliations:** ^1^ Department of Medicine and Cancer Center, Howard University College of Medicine, Washington DC, USA; ^2^ Department of Pathology, Howard University College of Medicine, Washington DC, USA; ^3^ Department of Medicine and Cancer Center, Yale University, New Haven, CT, USA; ^4^ Cancer Biology Research Program, The University of Arizona Cancer Center, Tucson, AZ, USA; ^5^ Division of Gastroenterology, Department of Internal Medicine, University of Michigan, Ann Arbor, MI, USA

**Keywords:** MSI, colorectal cancer, African Americans, Hispanics, Caucasians

## Abstract

**PURPOSE:**

African Americans (AA) are at a higher risk of colorectal cancer (CRC) and some studies report a higher frequency of microsatellite instability (MSI) in this population while others report lower frequency compared to Caucasians.

**AIM:**

To determine and evaluate the association of race and clinical factors with MSI frequency through meta- analysis.

**METHODS:**

Twenty-two studies out of 15,105 (1997-2015) were evaluated after a search in different literature databases, using keywords “colorectal cancer, microsatellite instability, African Americans, Caucasians and Hispanics”. We used random effect meta-analysis to calculate the MSI frequency in all studies as well as in African American and Caucasian samples. Meta-regression analysis was used to assess the univariate effect of race, gender, age, tumor location and stage on MSI frequency.

**RESULTS:**

The overall MSI frequency among CRCs was 17% (95%CI: 15%-19%, I²=91%). In studies with available race data, The MSI rate among AAs, Hispanics and Caucasians were 12%, 12% and 14% respectively and was not significantly different. Sub-group analysis of studies with racial information indicates MSI OR of 0.78 for AAs compared to Caucasians.

**CONCLUSION:**

CRCs demonstrate an overall MSI frequency of 17%. MSI frequency differences between AAs and Caucasians were not pronounced, suggesting that other factors contribute to the racial disparity. The methodological approaches and biological sources of the variation seen in MSI frequency between different studies need to be further investigated.

## INTRODUCTION

Colorectal cancer (CRC) remains a significant cancer burden and is the third most common cause of cancer-related deaths in the US. African Americans (AA) have the highest incidence and mortality rates of colorectal cancer. Despite significant improvement in screening approaches contributing to a lower annual prevalence and declining mortality rate in Caucasians, the mortality rate in AAs has not decreased to the same extent [ 1]. Although many factors correlate to the observed health disparity between African American and Caucasian patients, genetics and tumor biology may be an important contributor [2–7].

Colorectal cancer arises through a multi-step process in which genetic and epigenetic alterations accumulate in a sequential manner. Three different pathogenic pathways have been implicated. The majority (85%) display chromosomal instability. A minority (15%) of tumors, having a better prognosis, progress through the microsatellite instability (MSI) pathway and is associated with inactivation of DNA Mismatch Repair (MMR) genes. A third group of tumors, somewhat overlapping with MSI tumors, undergoes aberrant methylation and display the CpG island methylator phenotype (CIMP) [4, 8].

African Americans with CRC are typically diagnosed at a younger age than Caucasians [9, 10] and display high mortality rates even at early stages of CRC [2, 3] In addition, AA have a higher occurrence of proximal tumors than Caucasians for reasons that are unknown [6, 9–11].

Many epidemiologic and genetic investigations have focused on AAs [2–4] with a goal of interpreting the reasons for such disparities. Whereas one cannot discount low socioeconomic status for a more advanced stage of disease at diagnosis in AAs, other potentially modifiable risk factors like physical inactivity, smoking, unhealthy diet and obesity also contribute to significant proportion in new onset CRC [5]. Another important determinant of outcome in different racial groups is screening for CRC and it lags in AA compared to Caucasians irrespective of whether endoscopy or fecal occult blood testing was the modality. Data also indicates that AAs were less likely to have follow-up colonoscopy within 1 year of an abnormal flexible sigmoidoscopy examination compared to Caucasians [12].

MSI is a biomarker for detecting defective DNA MMR in CRCs. It is typically assessed by analyzing at least five microsatellite markers: three dinucleotide (D2S123, D5S346, D17S250) and two mononucleotide (BAT25 and BAT26) repeats referred to as the National Cancer Institute (NCI) consensus panel [5]. However, some limitations due to the use of dinucleotide markers show lower sensitivity and specificity compared with mononucleotide markers [13]. Hamelin et al [13] suggested a new panel of five quasi-monomorphic mononucleotide markers, known as the pentaplex panel, which revealed fairly accurate identification of MSI tumors without the need of matched normal DNA [14]. Three levels of MSI can be identified: high- level MSI (MSI-H), generally defined as MSI in more than 30% of the standard markers; low level MSI (MSI-L), when changes exhibited in less than 30% but greater than 0 % of the markers and microsatellite stable (MSS) in the absence of any microsatellite alterations. Mononucleotide repeat markers have been shown to be highly sensitive in detecting MSI-H tumors. Indeed, the use of BAT 26 alone has been found to be highly correlated with the NCI panel [6].

Defective DNA MMR within a CRC depicts improved prognosis for patients as compared to patients with proficient DNA MMR. Additionally, CRCs with defective MMR are hypermutated, often proximally located in the colon, and demonstrate enhanced immune reactions triggered by frameshifted neoantigens [15].

To gain better insight into the clinical usefulness of MSI, we have undertaken a systemic review of published studies, used meta-analysis techniques to derive a more precise, updated estimate of MSI frequency. We evaluated and compared different ethnicities (AA, Caucasian and Hispanic), demographic, and clinicopathological data in relation to MSI status to inform an approach to clinical care in these population.

## RESULTS

### Clinical characteristics showed higher rates of stages III/IV and right sided tumors

**Figure 1:**
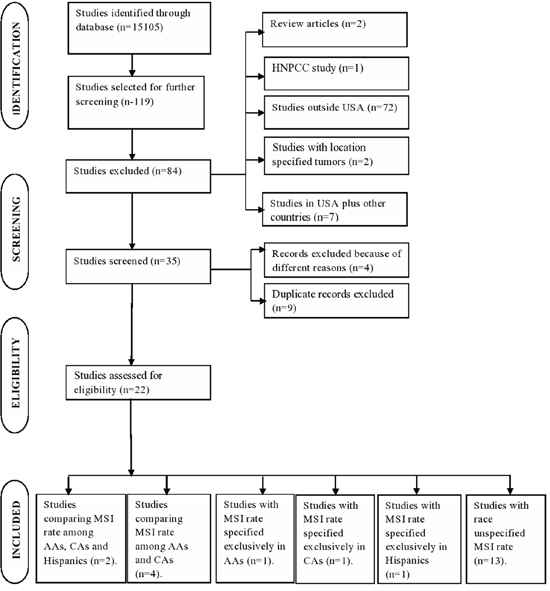
Flow chart of study selection process

Twenty-two selected studies [1, 16–36] analyzed a total of 12,611 colorectal patients according to MSI status (Figure 1 and Table 1). The median number of patients was 369 per study (range 61-2,720). Table 2 represents the characteristics of the patient population.

**Table 1:** Summary of studies: MSI frequency in colorectal cancer

STUDY	NUMBER OF PATIENTS	CRC TERMED AS	MSI TERMED AS	N POSITIVE MARKER THRESHOLD TO ASSIGN MSI-H	MSI DETECTION BY PCR	MSI DETECTION BY IHC	MSI DETECTION BY IHC/PCR	N MARKERS USED	MSI %
**ASHKTORAB, DC 2009 [36]**	**222**	**COLORECTAL, COLON CANCER**	**MSI**	**>2 MARKERS**	**YES**	**NO**	**NO**	**5**	**19.8**
**SYLVESTER, IL 2012 [1]**	**371**	**COLORECTAL, CRC**	**MICROSATELLITE INSTABILITY, MSI**	**>30%**	**YES**	**NO**	**NO**	**5**	**13**
**CARETHERS, NC 2014 [19]**	**503**	**COLON CANCER**	**MSI, MICROSATELLITE INSTABILITY**	**>2 MARKERS**	**YES**	**NO**	**NO**	**5**	**11**
**XICOLA, IL 2014 [18]**	**635**	**COLORECTAL**	**MSI**	**>2**	**YES**	**NO**	**NO**	**6**	**9**
**SINCROPE, OH 2015 [20]**	**2720**	**COLON, CRC**	**dMMR**	**>1**	**PCR**	**NO**	**NO**	**NOT GIVEN**	**10**
**SLATTERY, MN AND CA 2000 [21]**	**1510**	**COLON CANCER**	**MSI**	**>2**	**YES**	**NO**	**NO**	**12**	**17**
**CARETHERS, CA 2004 [17]**	**204**	**COLORECTAL**	**MSI**	**>2 MARKERS**	**YES**	**NO**	**NO**	**5**	**17.6**
^*^GOLOGAN, PA 2005 [22]	**75**	**COLORECTAL CANCER, CRC**	**MICRO-SATELLITE INSTABILITY, MSI**	**>2**	**YES**	**NO**	**NO**	**5**	**23**
**GUPTA, TX 2010 [23]**	**111**	**COLORECTAL, CRC**	**MSI**	**>2 MARKERS**	**YES**	**YES**	**YES**	**5**	**9**
^*^CUSHMON, TN 2009 [24]	**111**	**COLORECTAL**	**MSI**	**>2 MARKERS**	**YES**	**YES**	**YES**	**5**	**18.9**
^*^IMAMURA, MA 2014 [35]	**1267**	**COLORECTAL**	**MSI**	**NOT AVAILABLE**	**NOT AVAILABLE**	**NOT AVAILABLE**	**NOT AVAILABLE**	**NOT AVAILABLE**	**15**
^*^ALEXANDER, USA 2001 [25]	**323**	**COLORECTAL CANCER, COLON CANCER**	**MICROSATELLITE INSTABILITY**	**>30%**	**YES**	**NO**	**NO**	**10**	**28**
^*^KIM, USA 2007 [27]	**542**	**COLORECTAL, COLON CANCER**	**MSI**	**>30%**	**YES**	**NO**	**NO**	**5**	**18**
^*^WATANABE, USA 2001 [28]	**516**	**COLORECTAL CANCER, COLONCANCER**	**MICRO-SATELLITE INSTABILITY, MSI**	**>30 PERCENT, SHIFT OF BAT26 MARKER**	**YES**	**NO**	**NO**	**10**	**19.7**
^*^HALLING, MN 1999 [29]	**508**	**COLORECTAL**	**MICRO-SATELLITE INSTABILITY, MSI**	**>30%**	**YES**	**NO**	**NO**	**11**	**15**
^*^HAMILTON, TX 2013 [30]	**1253**	**COLORECTAL, CRC**	**MICRO-SATELLITE INSTABILITY, MSI**	**NOT AVAILABLE**	**NOT AVAILABLE**	**NOT AVAILABLE**	**NOT AVAILABLE**	**NOT AVAILABLE**	**15**
**CHAO, NM 2000 [31]**	**201**	**COLORECTAL, COLON CANCER**	**MICRO-SATELLITE INSTABILITY, MSI**	**>2**	**YES**	**NO**	**NO**	**10**	**18**
^*^HERFARTH, MO 1997 [32]	**61**	**COLORECTAL**	**RER+, MICROSATELLITE INSTABILITY**	**>2**	**YES**	**NO**	**NO**	**7**	**21**
^*^GOEL, CA 2004 [33]	**173**	**COLORECTAL, CRC**	**MSI**	**>2**	**YES**	**NO**	**NO**	**5**	**20**
^*^GARRITY, MN 2004 [16]	**366**	**COLORECTAL, CRC**	**dMMR**	**>1**	**NO**	**YES**	**NO**	**2**	**14.8**
^*^SHIA, NY 2003 [34]	**216**	**COLORECTAL**	**MSI**	**>30%**	**YES**	**NO**	**NO**	**5**	**35**
^*^BERTAGNO LLI, NC 2009 [26]	**723**	**COLON CANCER**	**dMMR**	**>50%**	**YES**	**YES**	**YES**	**5**	**14.8**

* Race unspecified MSI rate (n=13)

**Table 2:** Demographic and clinical characteristics of patients

	N^*^	% Median (IQR)^**^
Male	18	53 (50-56)
Age over 60 years	14	61 (54-67)
African American	7	9 (7-54)
Distal colon tumor	14	46 (41-56)
Stage 3 and 4	12	53 (41-70)
Poor differentiation	15	20 (10-24)
Mucinous tumor	9	13 (11-34)
Kras mutation	6	31 (28-40)
Braf mutation	6	9 (7-14)

* Number of studies which reported the corresponding characteristics

** Based on all patients in each study (IQR=Interquartile range).

The percentage of males were found in 18 studies, with the median of 53% [1, 17-29, 31, 34-36]. Our data also showed that 61% of the population were older than 60 years (Table 2). In our reviewed papers, only 7 studies indicated AA percentage, with the median of 9% and IQR of 7-54 AA suggesting large heterogeneity within the studies [1, 17-21, 36]. Fourteen studies identified colonic location of tumors, with a median of 46% in the distal colon. Only 12 studies provided staging with a median of 53% of them at stage III and IV [1, 16, 18-20, 24, 27-30, 35, 36]. Poor differentiation was found only in 15 studies with a median of 20%, suggesting more than 70 percent patients belonged to low grade type of tumor [1, 16-18, 20, 25-31, 34-36]. Mucinous type of histology was found only in 9 studies with a median of 13% and IQR of 11-34 [17-19, 22, 25, 26, 31, 34, 36]. We also analyzed KRAS mutation in 6 studies with median of 31% and IQR of 28-40 suggesting a significant association of CRC patients with KRAS in our study [1, 18, 21, 24, 35, 36]. BRAF mutation was identified in a studies with median of 9% and IQR of 7-14 indicating no significant association [1, 18, 24, 30, 35, 36].

**Figure 2:**
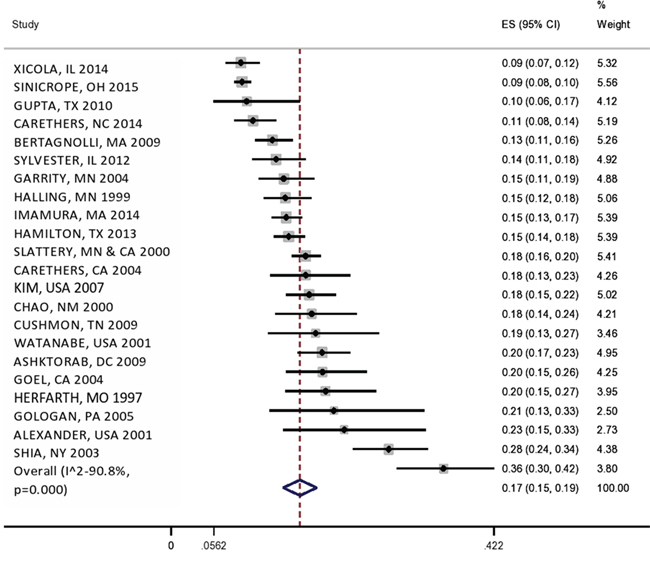

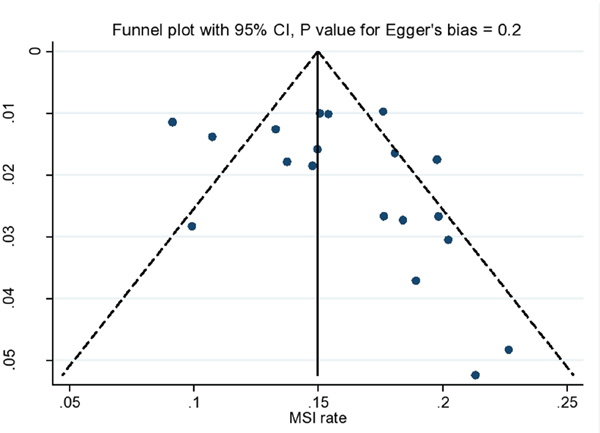
Figure 2a: Forest plot of MSI frequency (X-axis) by study (name of author, state and year). Figure 2b: Funnel plot showing the association between MSI% on X-axis and standard error on Y-axis for individual studies.

The mean (95%CI) of MSI percentage in all studies were 17% (15%-19%) with a heterogeneity of I^2^=91% (Figure 2a). Furthermore, we assessed the influence of removing each study on overall estimates which determined one study (MSI rate= 9%, N = 2,720) to have a significant influence. We also analyzed the effect of publication bias on overall estimates, which indicated a publication bias toward larger studies with higher MSI percentage, suggesting two influential outlier studies. By removing these two studies, the Egger's test for publication bias supports lack of bias (P = 0.2, Figure 2b). The overall MSI percentage in remaining 19 studies was 16% (14%-17%, I^2^=76%).

### Relationship between patients' characteristics and MSI percentage

We did meta-regression analysis to evaluate the patients' characteristics on MSI percentage (Table 3) and reduced the heterogeneity of results.

**Table 3:** Effect of patients' characteristics on MSI percentage and heterogeneity of results from meta-regression analyses

Predictor	Mean effect on MSI (P value)^*^	Adjusted I^2^
Year of study (each 5 years)	−0.02 (0.005)	61%
Male	−0.04 (0.8)	81%
Age over 60 years	−0.08 (0.2)	81%
African American	−0.01 (0.8)	83%
Distal colon tumor	−0.04 (0.6)	78%
Stage 3 and 4	0.11 (0.08)	63%
Poor differentiation	−0.06 (0.7)	79%
Mucinous tumor	0.3 (0.1)	73%
KRAS mutation	0.3 (0.1)	80%
BRAF mutation	0.2 (0.6)	74%

* For 1% increase in predictor

Our data showed that the more recent the study, the lower MSI rate (P = 0.005). The MSI rate in studies earlier than 2006 [16, 17, 21, 22, 25, 28, 29, 31-34] was 17% (95%CI: 16-19%, I^2^=9%) compared to 14% (95%CI: 13-15%, I^2^=41%) in recent studies. Other predictors of higher MSI rate were advanced stage, mucinous tumor and KRAS mutation (all P value ≤ 0.01, Table 3). The MSI rate was 16% (95%CI: 14-17%, I^2^=13%) in studies which used >30% threshold and 15% (95%CI: 13-16%, I^2^=59%) in studies that used >1 marker.

### MSI percentage in different races

#### MSI frequency in African Americans with CRC

**Figure 3:**
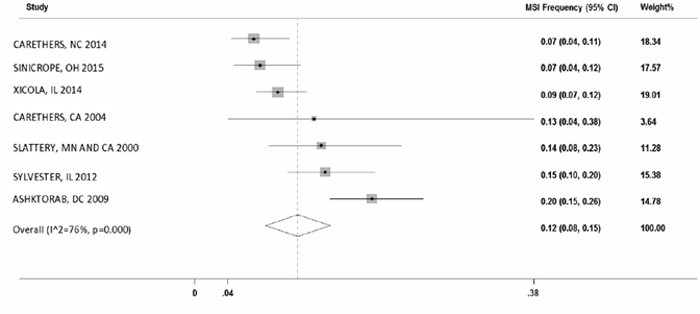
Forest plot of MSI frequency(X-axis) in African Americans

There were seven studies which reported MSI frequency in African Americans (n=1,349). The overall MSI percentage (95% CI) in these studies was 12% with I^2^ = 76% (8%-15%; Figure 3).

#### MSI frequency in Caucasians with CRC

**Figure 4:**
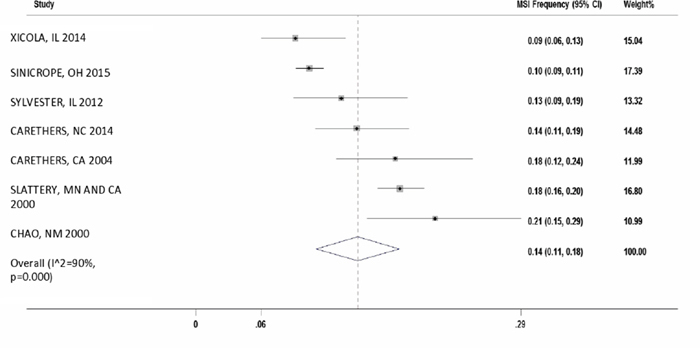
Forest plot of MSI frequency(X-axis) in Caucasians

There were seven studies which reported the MSI frequency specifically in Caucasians (n=4,642). The overall MSI percentage (95% CI) in these studies was 14% with I^2^ = 90% (11%-18%; Figure 4).

#### Difference of MSI frequency in African Americans and Caucasians

**Figure 5:**
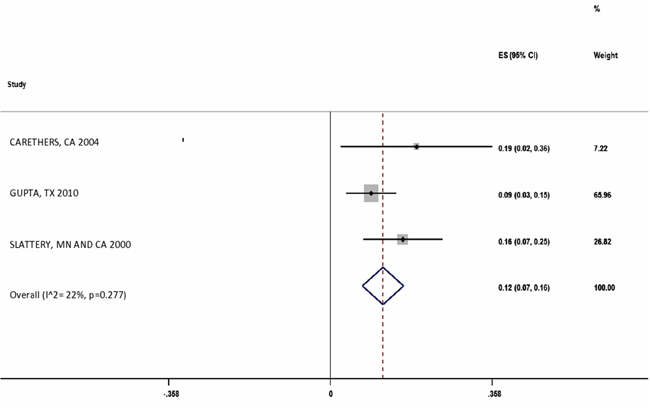
Forest plot of Odds Ratio of MSI frequency in African Americans compared to Caucasians

In six studies, we were able to calculate the odds ratio for MSI frequency in African Americans compared to Caucasians. The overall odds ratio (95% CI) was 0.78 (0.58-1.06) for AAs with I^2^ = 23%. (Figure 5).

#### MSI% and frequency in Hispanics with CRC

**Figure 6:**
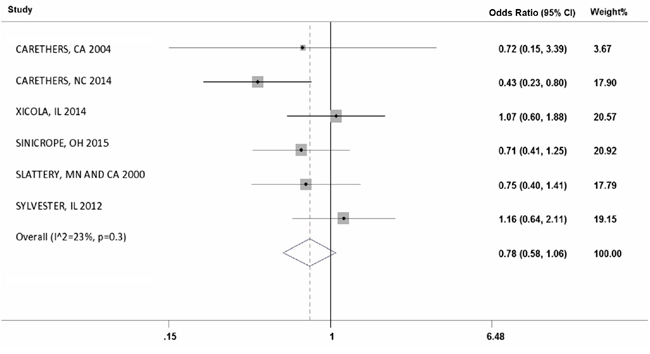
Forest plot of MSI frequency(X-axis) in Hispanics

There were three studies which reported the MSI frequency in Hispanics (n=203). The overall MSI percentage (95% CI) in these studies was 12% (7%-16%) with I^2^=22% (Figure 6).

## DISCUSSION

Microsatellite instability is a biomarker of genomic alteration from an impaired DNA mismatch repair system (DNA MMR). The consequences of faulty DNA MMR in CRC include improved survival compared with same staged patients with MMR-proficient tumors, improved response to PD-1 blockade and resistance to 5-FU. These consequences have implications for patient care. In our meta-analysis of the 22 studies within US, the overall MSI frequency was 17%. Thus, we would expect that 17% of CRC patients would show prolonged survival over patients with MMR-proficient CRCs.

The overall highest MSI-H frequency was found in New York and the lowest in North Carolina for all races, falling within the range of 7-34%. MSI frequency in AAs was highest in Washington D.C. (19%) and lowest in North Carolina (7%). This different range may be due to population heterogeneity and different environmental exposures and etiologies. This frequency also corresponds to the observed percentage of hypermutated tumors [37]. Though biology might play a role, the interaction between external (high dietary intakes of animal products) and internal (potentially toxic hydrogen producing bacteria and secondary bile salts) environments also determine the CRC risk [38]. In addition to this are technical artifacts associated with using both mononucleotide and dinucleotide markers or just mononucleotide markers or using PCR/IHC for analyzing MSI [14].

In order to get better insight, we analyzed 1,349 AA CRC subjects and 4,642 Caucasian CRC subjects in seven selected studies with mean MSI of 12% and 14%, respectively. This population-based study demonstrates the novel finding that MSI frequency in colon cancers from African American patients have almost same frequency compared to Caucasians. Our meta-analysis showed a large heterogeneity (I^2^ of 76% in AA vs. 90% in Caucasians) within the studies.

Only a portion of the 22 studies had a differentiation of CRC patients by race. The determined numbers of AAs in our meta-analysis from the seven studies with AA data should provide overall insight into an average MSI frequency in this population. Indeed, AAs showed 12% MSI frequency in their CRCs, while Caucasians had 14% MSI frequency. In comparing six studies in which both of these were identified, the OR for MSI in AA was 0.78 meaning AAs were less likely to have MSI in their tumors. The overall MSI frequency among Hispanics was 12%, but there were not clear studies in which to do direct comparisons in Caucasians to determine an odds ratio. Although MSI CRCs are 17% of the total, the lesser frequency among AAs may be one contributor towards the disparity of CRC survival.

MSI frequency among races was not statistically significant (P>0.9) suggesting that there is a great variability in MSI-H status of CRC patients across the USA, underlining the need for robust prognostic and predictive molecular markers. MSI-H CRCs have been reported to demonstrate more frequent association with proximal tumor location, poorly differentiated, mucin-containing histology and advanced staging [39]. Many of the observations made in this study regarding the clinicopathological features of sporadic MSI-H tumors are in concordance with previously published studies.

BRAF and KRAS mutations occur in about 15% and 35% of sporadic CRCs, respectively. The proto-oncogene BRAF is a member of RAF kinase family of growth signal transduction. It activates the MAP kinase/ERKs signaling pathway, which affects cell division and differentiation. The proto-oncogene KRAS is a member of RAS gene family and is a downstream effector of the EGFR gene in the RAS-RAF-MEK-ERK signaling pathway. The RAS-RAF-MEK-ERK signaling pathway is commonly involved in cell cycle progression and cell proliferation, and thus, activating mutations of key component genes in this pathway, including mutations in the BRAF or KRAS gene, can bring about uncontrolled cell growth and increased cell survival and may play an important role in tumorigenesis. It has been revealed that BRAF V600E mutations are highly associated with MSI-H CRCs, although the incidence of KRAS mutations is inversely correlated with MSI-H status in CRCs [6].

We also analyzed KRAS mutation in six studies with median of 31% and IQR of 28-40 suggesting significant association of CRC patients with KRAS in our study and in another study (Study #2). African Americans with microsatellite stable (MSS)/MSI-low (MSI-L) tumors have a higher proportion of KRAS mutations than Caucasians [1, 18, 21, 24, 35, 36]. The question arises as to whether it is worth investigating this association in the clinical setting. Considering that KRAS mutations may also confer resistance to EGFR inhibitors, patients who have colorectal cancer with KRAS mutation could receive more tailored management. A limitation of this meta-analysis is only six studies investigated KRAS mutation, as further studies are necessary to answer important questions and a large sample size is a pre-requisite for assessing the association of KRAS mutation with MSI along with other tumor molecular characteristics. In addition, in our meta-analysis, only six studies reported BRAF mutation and it did not show any association between MSI-H and BRAF mutation [1, 18, 24, 30, 35, 36]. Additional studies need to be adequately powered to do so. As an example, this may require racial- and/or ethnic-specific enrollment, such that a trial could close to accrual for whites but remain open for African Americans until adequate accrual is reached; or trials should be opened to a large global community of different races/ethnicities.

We conclude that the MSI frequency among CRC tumors is 17% although we calculate an OR of 0.78 for AA MSI CRCs compared to Caucasian MSI CRCs, statistics show an insignificant P value, indicating no difference in frequency. Despite this, individual studies generally have low numbers of AA CRC patients in their cohorts, contributing to wide intervals for MSI frequency. Further studies should accrue adequate numbers of AA patients to assess the impact of MSI on their care.

## MATERIALS AND METHODS

### Search strategy

Studies from 1997 to 2015 were searched using following data bases: PubMed, Google scholar, Science direct, Scopus, Cross references and Citations using keywords; “colorectal cancer” or “colon cancer” combined with any of “microsatellite instability”, “MSI”, “Replication error”, “Mismatch repair”, “dMMR”, “loss of Heterozygosity” combined with any race “African Americans”, “Caucasians” and “Hispanics.” All studies matching eligibility criteria were retrieved and bibliographies were reviewed. Our inclusion criteria included study populations belonging to USA and English language publications that provided estimates of MSI percentage and/or number among CRCs. We included studies assessing MSI by analyzing DNA molecular markers for MSI and/or IHC. We excluded in vitro models articles, or the studies that did not have detailed demography or MSI, case reports and repetitive publications. All overlapping and duplicated databases were removed.

### Data extraction and study assessment

For each study the following data were extracted: The first author surname, state of origin (USA), publication year, sample size, number of MSI, MSI percentage, race specified MSI CRCs, age, gender, tumor location, staging, tumor grade, KRAS mutation frequency rate, BRAF mutation frequency rate, presence of mucinous tumor, colon vs. rectal carcinoma.

### Reviewed studies

After reviewing 15105 studies, 119 were selected for further screening based on their specific assessment of MSI within colorectal cancer patients. Of these 119 studies, 97 were removed because they corresponded to non-US population (n=72, mixed groups with no clear geographic location (n=7)), review studies (n=2), studies from hereditary non-polyposis colorectal cancer patients (HNPCC; n=1), studies from appendix and rectal cancer patients (n=2), studies where patients were selected based on their MSI status (n=4) and duplicate studies of which the patients were included in recent publications by the same authors (n=9). Therefore, there were 22 studies from 1997 to January 2015 that were included for systematic review and meta-analysis [1, 16–36].

### Staging of tumors

In our retrieved papers, different tumor staging systems were used. In order to make them comparable Duke's and Astler-Collar stages were transformed into TNM staging systems. In two studies, staging was omitted because it was described as local and distant and therefore not convertible to TNM staging.

### Microsatellite instability status & number of markers used

Established dinucleotide and mononucleotide microsatellite markers was used for MSI status assignment within our review and Meta-analysis (Table 1). The median number of genotyped markers were five (range 2-12). Two studies analyzed only dinucleotide markers, whilst 2 studies considered only mononucleotide markers and 2 studies classified MSI status by both DNA microsatellite markers and by IHC analysis. Only 1 study assigned MSI status exclusively by IHC. Most studies divided patients into MSI-H and MSS. In such cases, MSS group included both MSI-L and real MSS patients.

### Statistical analysis

The overall MSI frequency in all studies was calculated from a random effect meta-analysis for a binary variable. The I^2^ for heterogeneity of effect between different studies were calculated. We assessed the effect of summary patients' characteristics on the frequency of MSI with meta-regression. Also we assessed the frequency of MSI in African American, Caucasian and Hispanic groups separately. The odds ratio (95% CI) of association between race (African American vs. Caucasian) and MSI frequency was used for a separate meta-analysis to assess the overall effect of race on MSI frequency. We used funnel plots to assess the publication bias and the meta-influence analysis to explore the influence of individual on summary effect. All analyses were performed in Stata 14.0 (Stata Corp, College Station, TX).
